# Neuroplasticity Mechanism of Stroke Rehabilitation Training System Based on Virtual Reality: A Review

**DOI:** 10.3390/s26061753

**Published:** 2026-03-10

**Authors:** Runzi Cheng, Hui Xu, Xing Wang

**Affiliations:** 1 Department of Biomedical Engineering, Chongqing University, Chongqing 400044, China; 20233388@stu.cqu.edu.cn (R.C.); 202519021048t@stu.cqu.edu.cn (H.X.); 2 Department of Biomedical Engineering, The Key Laboratory of Biorheological Science and Technology, Ministry of Education, Bioengineering College, Chongqing University, Chongqing 400044, China

**Keywords:** virtual reality, stroke, rehabilitation training, neuroplasticity

## Abstract

**Highlights:**

**What are the main findings?**

- Virtual reality (VR) technology shows significant application potential in upper-limb function recovery, lower-limb gait balance rehabilitation, and cognitive rehabilitation for stroke patients, and these approaches can induce cerebral functional reorganization via task-oriented training and adaptive feedback mechanisms.
- Multimodal neuroimaging techniques, namely electroencephalography (EEG), functional magnetic resonance imaging (fMRI), and functional near-infrared spectroscopy (fNIRS), can non-invasively quantify neuroplasticity changes induced by VR intervention.

**What are the implications of the main findings?**

- The VR-based stroke rehabilitation training system is associated with the mechanism of neuroplasticity, which provides important theoretical support for the development of personalized and accurate clinical rehabilitation programs.

**Abstract:**

The paper systematically reviews the application status of virtual reality technology in the rehabilitation of upper-limb movement, lower-limb gait balance, and cognitive function of stroke patients. Based on electroencephalography (EEG), functional magnetic resonance imaging (fMRI), and functional near-infrared spectroscopy (fNIRS), the correlation mechanism of virtual reality promoting brain functional reorganization and neural remodeling is analyzed from the perspective of task-oriented training, reinforcement learning, and neural regulation. The virtual reality rehabilitation scheme can accurately match the actual needs of clinical rehabilitation, and exploring the internal mechanism of its intervention in the dynamic process of rehabilitation is helpful to promote the deep integration of virtual reality technology and rehabilitation medicine. This study integrates high temporal resolution EEG activity data, magnetic resonance imaging spatial positioning information, cerebral hemodynamic data, and virtual reality system behavior data, realizing the systematic quantitative output of rehabilitation effect in the “human-computer” interactive closed loop. Finally, the future development direction is projected from the aspects of system optimization, standard setting, and multi-technology integration to provide a reference for promoting the clinical application and development of virtual reality technology in stroke rehabilitation.

## 1. Introduction

Stroke, also known as cerebrovascular accident, refers to a disease that causes acute brain dysfunction due to sudden stenosis, occlusion, or rupture of intracerebral arteries. This disease is characterized by a high incidence rate, a high disability rate, and a high mortality rate [1]. According to statistics, stroke is the largest contributor to disability-adjusted life years (DALYs) in the global nervous system. From 1990 to 2016, the global incidence rate of stroke increased by 78%, and the health impact increased by 22% [2]. Stroke may cause various problems, such as motor dysfunction [3], speech impairment [4], and cognitive impairment [5], directly affecting patients’ daily lives [6]. Rehabilitation training has become a necessary approach for them to regain control of body movements and improve their quality of life [7]. However, traditional rehabilitation training is monotonous and lacks interest, resulting in poor patient compliance, low treatment enthusiasm, and low cooperation [8]. Moreover, it relies too much on therapist experience, making it difficult to standardize training intensity, frequency, and intervention strategies, leading to significant individual differences in treatment efficacy. These practical difficulties urgently require technological innovation breakthroughs to provide new paths for improving the functional rehabilitation outcomes of stroke patients. With technological advancements, virtual reality technology has developed rapidly and is gradually being applied in the field of stroke rehabilitation.

Virtual reality (VR) technology is defined as “an interactive simulation created using computer hardware and software, providing users with the opportunity to participate in an environment similar to real-world objects and events [9]”. It creates a realistic 3D environment for users through head-mounted devices (such as VR glasses), sensors, and other equipment, featuring three core characteristics: immersion, interactivity, and imaginativeness. The advantages of VR training, such as controllability, repeatability [10], and deep immersion [11], have demonstrated great potential in the field of rehabilitation medicine. In 2008, the European Stroke Organization (ESO) first proposed the use of virtual reality training technology for stroke patients with sequelae to improve their motor function [12], marking the authoritative recognition of the clinical value of VR rehabilitation technology. Since the 21st century, VR-based stroke rehabilitation training has achieved some promising results. For example, in 2020, the Swiss company Hocoma [13] developed a virtual reality system called ArmeoSpring for upper-limb rehabilitation after stroke, which can assist patients with joint movement while providing relevant motion parameter assessments. In 2023, Beijing Science and Technology Park [14] proposed a high-precision home rehabilitation system based on VR rehabilitation medicine and upper-limb rehabilitation robot technology, enabling patients to undergo rehabilitation training and obtain assessment results without therapists. Multiple meta-analyses have shown that VR can improve upper-limb and balance function in stroke patients to varying degrees [15], as well as the balance function in patients with Parkinson’s disease and multiple sclerosis [16].

Neuroplasticity refers to the brain’s ability to reorganize itself by forming new neural connections in response to learning, experience, and injury [17]. In the virtual environment constructed by VR, there is a dynamic interaction between the patient’s sensory input, motor response, and cognitive engagement, which in turn triggers a series of neuroplasticity changes, reflecting the therapeutic potential of VR in neurorehabilitation [18]. However, current research on the application of VR in stroke rehabilitation training focuses on rehabilitation outcomes, often neglecting the underlying principal mechanism centered on neuroplasticity. This article systematically elaborates on the application of VR in the rehabilitation of upper and lower limbs and cognitive function in stroke patients. Through a series of neurofunctional assessment methods, it explores the principal mechanism behind VR rehabilitation training based on neuroplasticity, fills the gap in relevant literature regarding the theoretical basis of VR rehabilitation technology, and provides practical examples for reference to relevant researchers and clinicians, pointing out the direction for the future development of VR-based stroke rehabilitation training.

## 2. Virtual Reality Rehabilitation Training System Architecture

The core design of the VR rehabilitation training system lies in constructing a complete closed-loop human-computer interaction consisting of “perception–processing–interaction–feedback”. It provides standardized engineering support for rehabilitation training of stroke patients and neuroplasticity correlation research. The closed-loop system is composed of four core modules: perceptual input, data processing, virtual reality interaction, and multimodal feedback regulation. The instrument parameter configuration of each module directly affects the success rate of training and neuroplasticity results. The specific combination of the closed-loop characteristics and the impact of instrument parameters is as follows.

### 2.1. Closed-Loop System Architecture

As the starting point of the closed loop, the core function of the perceptual input module is motion sensing. The module involves motion capture [19], electromyography, EEG, and other technologies to provide the basis for subsequent processing. The data processing module completes signal processing through a specific algorithm to achieve relevant feature extraction and improve the accuracy of the system [20]. The virtual reality interaction module constructs an immersive training scene to realize the precise linkage between the patient and the system. The multimodal feedback regulation module transmits patients’ training information and physiological signals to guide the medical staff to optimize the scheme. The new signal is input into the perceptual module again to form a complete iteration [21]. The collaborative operation of the four modules directly affects the rehabilitation effect and neuroplasticity by affecting the signal quality, interactive experience, and control accuracy.

### 2.2. Influence of Instrument Parameters on Rehabilitation Outcome

(1) Sampling rate

The sampling rate determines the reduction accuracy of the collected signal. It affects the accuracy of rehabilitation and the reliability of neuroplasticity analysis. Motion signal acquisition includes Kinect (30 fps), and neurophysiological signal acquisition includes EEG (500 Hz) [22], fNIRS (10 Hz) [23], etc. An insufficient sampling rate will lead to signal distortion and loss of key features, while too high a sampling rate will increase data redundancy and processing delay.

(2) Spatial resolution

Spatial resolution affects the accuracy of neural signal localization and virtual scene presentation, which is crucial for neuroplasticity analysis and training compliance. EEG and fNIRS need to ensure high spatial resolution to accurately locate the brain activation area. Taking the Kinect V2 as an example, the spatial resolution of the head-mounted display is 1920 × 1080 to ensure the clarity of the scene. At the same time, it is necessary to balance the spatial resolution and rendering delay to avoid damaging the human–computer synchronization.

(3) Feedback delay

Feedback delay is the key factor affecting the closed-loop fluency, which directly determines the training effect and the formation of neuroplasticity. The total delay of multimodal feedback should be less than 50 ms (including sampling delay, processing delay, control delay, command transmission delay, etc.) [24]. If the delay is more than 50 ms, it will destroy the human–computer cooperation, hinder the reinforcement learning and neural function reorganization, and reduce the training compliance.

(4) Tactile rendering accuracy

Tactile rendering accuracy affects interactive authenticity, which is of great significance for fine motor rehabilitation. Taking the upper-limb rehabilitation as an example, the fine motor rehabilitation needs to meet the accuracy (0.4 N) [25] and flexibility (tactile cycle execution time 1 ms) [26] of the tactile feedback device to reconstruct the relationship between tactile and motor control and promote the synergistic activation of the cerebral cortex. Precise tactile rendering can also improve the patient’s training initiative and provide continuous stimulation for neuroplasticity.

It should be noted that at present, there is no complete clinical experimental data to confirm the optimal instrument parameters, and the above are only the results of some experiments.

To sum up, the smooth operation of the human–computer closed loop of the VR rehabilitation system depends on the optimization of key instrument parameters. The settings of parameters are directly related to the rehabilitation effect and the outcome of neuroplasticity by affecting the degree of signal reduction and interactive experience. Reasonable configuration of parameters can provide standardized support for rehabilitation training and neuroplasticity correlation analysis.

## 3. Current Application of VR in Rehabilitation Training for Stroke Patients

**Figure 1:**
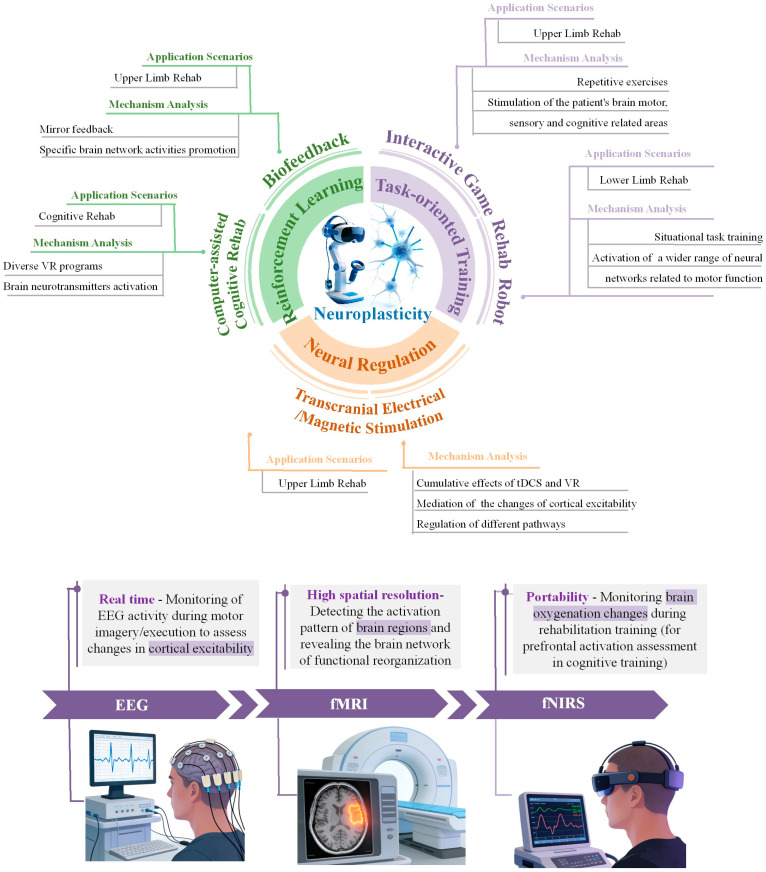
Framework of neuroplasticity regulation and multimodal neuroimaging assessment for virtual reality in stroke rehabilitation. (**a**) Core mechanisms and application scenarios. Centered on neuroplasticity, a multimodal VR rehabilitation intervention system is constructed by integrating five key technical pathways: (1) biofeedback; (2) rehabilitation robot; (3) transcranial electrical/magnetic stimulation; (4) interactive game; and (5) computer-assisted VR programs. The system can be applied to upper-limb motor, lower-limb gait/balance, and cognitive function rehabilitation scenarios. (**b**) Comparison of multimodal neuroimaging technology characteristics. For monitoring neural activity during VR rehabilitation, three mainstream technologies exhibit distinct advantages: (1) electroencephalography (EEG) enables millisecond-scale real-time monitoring to capture cortical electrical activity changes during motor execution; (2) functional magnetic resonance imaging (fMRI) offers high spatial resolution, allowing precise localization of activated brain regions; and (3) functional near-infrared spectroscopy (fNIRS) provides portability, enabling continuous monitoring of cerebral hemodynamics to deliver objective evidence for rehabilitation outcome assessment.

The research results are analyzed and summarized from two aspects: the current application status and the impact of neuroplasticity. The current application status includes upper-limb functional rehabilitation, lower-limb function and gait balance rehabilitation, and cognitive rehabilitation. The impact of neuroplasticity includes task-oriented training and functional reorganization, adaptive feedback and reinforcement learning, as well as neural regulation and excitability modulation. Table 1 provides a comprehensive overview of the neuroimaging analysis outcomes related to virtual reality rehabilitation in stroke patients. The framework of neuroplasticity regulation and multimodal neuroimaging assessment for virtual reality in stroke rehabilitation is illustrated in Figure 1.

### 3.1. Upper-Limb Functional Rehabilitation

#### 3.1.1. Interactive Game

Since the early 2000s, there has been increasing interest in using sports games and virtual reality-based interventions as innovative methods to enhance physical rehabilitation for individuals with multiple disabilities [39]. Among these, interactive sports games specifically developed by researchers for stroke patients have demonstrated tangible benefits for upper-limb rehabilitation. Xiao et al. [40] developed a virtual reality rehabilitation system based on the visual capture sensor Kinect. Experimental results showed that the system’s motion angles were accurate and stable, and the virtual guidance effectively guided patients through preset exercises. To further improve VR-based sports games, Villada et al. [39] introduced an iterative approach, arranging a final meeting to test the overall functionality of the integrated system. The VR sports game for patients exhibited a medium-to-high level of game user experience, Villada thus validating the important role of iterative design methods in the development of rehabilitation games.

#### 3.1.2. Biofeedback

Over the past few decades, VR has been integrated into biofeedback (BF) therapy programs. Studies have shown that VR-BF may possess advantages in terms of motivation, user experience, engagement, and attention concentration [41]. Among these, visual and tactile feedback are most widely used in stroke rehabilitation. On the one hand, VR-based mirror visual feedback can provide enhanced stimulation and promote neuroplasticity during the rehabilitation process and has been applied to treat stroke [42], phantom limb pain [43], and complex regional pain syndrome. Patricia et al. [33] created a perceptual illusion of controlling the impaired virtual side through mirroring in the virtual environment. The results indicated that all datasets exceeded a System Usability Scale (SUS) score of 68, suggesting that the proposed task usability and visual feedback were satisfactory. On the other hand, tactile interaction plays a crucial role in VR systems, facilitating a more immersive virtual experience by enabling realistic interaction between the operator and virtual objects. It is also worth exploring for applications such as virtual training and virtual control [44]. Zhang et al. [45] introduced a design concept called TouchMark to explore the impact of partial tactile feedback provided by different tangible handle tools on user experience in virtual rehabilitation. The evaluation results showed that the presence of partial tactile stimulation significantly enhanced the user’s sensory feedback, which is consistent with existing research indicating that tactile feedback enhances the realism of interaction within the virtual environment [46,47].

#### 3.1.3. Transcranial Direct Current Stimulation

In recent years, transcranial direct current stimulation (tDCS), as a non-invasive brain stimulation technique, has been frequently applied to improve motor function in post-stroke rehabilitation [35]. Preliminary studies on individuals with mild impairment in the acute phase after stroke have shown that the effectiveness is enhanced when tDCS is combined with VR [37]. Both Lee et al. [48] and Yao et al. [36] explored whether the combination of tDCS and VR is superior to VR training alone in reducing motor dysfunction and improving upper-limb function in stroke patients. The results indicated significant improvements in the shoulder/elbow/forearm segments and coordination/speed components in the experimental group. Furthermore, when comparing the effectiveness of the combined intervention of tDCS and VR with conventional physical therapy, Llorens et al. [37] confirmed sustained improvements in the Fugl-Meyer Assessment Scale (FMA) scores (P < 0.001, $\eta_{p}^{2}$ = 0.44), Wolf Motor Function Test time (P = 0.036, $\eta_{p}^{2}$ = 0.15), and ability subscale (P = 0.043, $\eta_{p}^{2}$ = 0.14), which were different from the limited improvements observed in the conventional group. It was also found that the combined therapy could induce changes in cortical excitability by regulating the conductivity of sodium and calcium channels and the activity of NMDA receptors, thereby further modulating the neuroplasticity of patients.

### 3.2. Lower-Limb Function and Gait Balance Rehabilitation

#### Lower-Limb Rehabilitation Robot

Robot-assisted therapy (RAT) has been widely applied based on the principles of motor relearning and neuroplasticity. The introduction of VR provides multimodal stimulation and multisensory feedback during training, maximizing the afferent input of peripheral joints and providing specific task-based stimulation to the central nervous system [49]. Akinci et al. [50] explored the impact of a VR-based lower-limb gait training robot system on gait and balance function in stroke patients. Statistical parameters showed significant improvements in walking speed and walking distance in each group, indicating the effectiveness of lower-limb rehabilitation robots in improving balance after stroke [51]. Further combining the principles of motor learning and plasticity, Zhou et al. [52] developed a rehabilitation training system that integrates robotic movement assistance with neural circuit-based virtual reality (NeuCir-VR). The system enables programmed lower-limb rehabilitation training with a reward mechanism. Using functional magnetic resonance imaging (fMRI) data, the study found that this approach, through the multisensory feedback provided by virtual reality, can effectively promote the remodeling of neural motor circuits.

### 3.3. Cognitive Rehabilitation

#### Computer-Assisted Cognitive Rehabilitation

Computer-assisted cognitive rehabilitation (CACR) is a computer-based cognitive training program [53]. In advanced VR systems, multiple authors have emphasized the good effects of CACR on rehabilitation [54]. Kim et al. [55] compared the efficacy of combined VR and CACR therapy with that of CACR alone and found that the combined group showed significant improvements in visual attention and short-term visual–spatial memory in patients with cognitive impairment due to acute stroke. This is consistent with the finding that VR programs providing visual–spatial sensory stimulation training can significantly improve cognitive function in patients [56]. Based on comparing the improvements in cognitive and motor functions in stroke patients, Zhou et al. [57] demonstrated, through measuring the levels of neurotransmitters such as dopamine and the amplitude and latency of event-related potential P300, that combined intervention enhances brain sensitivity to stimuli, increases neurotransmitter levels, promotes neuroplasticity, and improves neurological dysfunction by providing multisensory comprehensive stimulation [58].

## 4. Correlation Analysis of VR and Neuroplasticity in Stroke Rehabilitation Based on Neuroimaging Technology

In summary, numerous clinical studies have shown that virtual reality-based rehabilitation training systems exhibit significant advantages in improving the upper and lower limbs as well as cognitive functions of stroke patients. However, the underlying reason for these observable behavioral improvements stems from the profound impact of VR intervention on the intrinsic neural remodeling process of the brain. Current research is no longer satisfied with merely studying rehabilitation functions but rather delves into how VR can serve as a “neurobehavioral intervention tool” to drive the reorganization of brain structure and function. Therefore, to understand the effectiveness of VR rehabilitation from a mechanistic perspective, researchers have begun to utilize various neuroimaging and electrophysiological techniques to directly or indirectly observe and verify the neuroplasticity changes induced by VR. Among these, electroencephalography (EEG), functional magnetic resonance imaging (fMRI), and functional near-infrared spectroscopy (fNIRS) constitute the three core research methods currently employed.

### 4.1. Methods for Verifying Neuroplasticity Induced by Virtual Reality

#### 4.1.1. Electroencephalogram

An electroencephalogram (EEG), as a non-invasive and portable neuroimaging method, is applied to extract the characteristics of neuroplasticity induced by VR rehabilitation methods. This method assesses the synchrony of cortical activity by analyzing the power spectral changes in rhythms such as θ, α, and β. Studies have shown that stroke patients who undergo VR cognitive training experience a significant increase in α power in the occipital region and β power in the frontal lobe region [59], as well as stronger event-related spectral perturbations in the high γ (46–70 Hz) and β bands [60], demonstrating the potential effectiveness of VR in promoting neuroplasticity.

#### 4.1.2. Functional Magnetic Resonance Imaging

Functional magnetic resonance imaging (fMRI) is a neuroimaging assessment technique used to study the effects of brain rehabilitation. It reflects changes in functional brain networks and neural activity by detecting concurrent fluctuations in blood oxygen level-dependent signals occurring in different brain regions [34]. It has been applied to the study of network topological changes induced by VR rehabilitation in stroke patients. Research has shown that VR-based limb rehabilitation training can promote cerebellar recruitment and prefrontal cortex activation, as well as changes in the frontal parietal network, strengthening connections between ipsilateral and interhemispheric frontal-parietal and motor cortices [34,61]. This demonstrates that VR rehabilitation training systems can promote neuroplasticity in motion-related areas.

#### 4.1.3. Functional Near-Infrared Spectroscopy

Functional near-infrared spectroscopy (fNIRS) is a non-invasive, flexible, and low-cost brain imaging technique [62]. Its excellent motion tolerance allows participants to undergo brain measurements while maintaining a certain level of activity, making it suitable for real-time brain function monitoring during VR training. Experimental results emphasize that during VR rehabilitation, patients exhibit an increased hemodynamic response of oxyhemoglobin and enhanced cortical activation (prefrontal cortex, premotor cortex, supplementary motor area, primary sensorimotor cortex, and somatosensory association cortex) [27,63], validating the induction effect of VR on cortical activation.

### 4.2. Correlation Mechanism Between VR Intervention and Neuroplasticity in Stroke Rehabilitation

#### 4.2.1. Task-Oriented Training and Functional Reorganization

(1) Interactive Game

The core of VR-based interactive games lies in simulating various functional tasks. This highly contextualized, motivation-driven repetitive practice stimulates the relevant areas of the brain responsible for movement, sensation, and cognition, thereby achieving the remodeling and optimization of task-based functional neural circuits [64]. The optimization results can be reflected by cortical activation levels and EEG. In terms of cortical activation, Bae et al. [27] measured cortical activation in stroke patients after training in a rhythm game using fNIRS and found that motor execution and sensorimotor integration activated the primary sensorimotor cortex, premotor cortex, and supplementary motor area, demonstrating the promotion effect of high cognitive load sensorimotor tasks on prefrontal cortex activation. Similarly, Bao et al. [28] used fMRI to evaluate the impact of Kinect virtual reality training on subacute stroke and found statistically significant cortical activation patterns in the contralateral primary sensorimotor cortex, bilateral supplementary motor areas, and ipsilateral cerebellum in all subjects. In terms of EEG, Lee et al. [29] measured brainwave activity in stroke patients before and after VR-based upper-limb training and found that tasks requiring focused attention reduced α waves and increased β waves. Meanwhile, bilateral upper-limb training based on VR significantly increased brain activity in the frontal pole 1 area and frontal 3 area, consistent with the principle that some task training can modulate more specific brain regions within the cortical motor area [65].

(2) Rehabilitation Robot

The integration of robotic training into VR scenarios has facilitated the transition from mere assistive movement to task-oriented training. This contextualized task training is closer to the demands of the real world, activating a broader range of neural networks related to motor function and significantly enhancing the effect of neural remodeling [52]. In terms of EEG, Calabrò et al. [30] investigated the neurophysiological basis of motor function recovery caused by Lokomat and VR. The results showed an increase in central μ/β power and a significant frontal–occipital high γ (46–70 Hz) event-related desynchronization, which is considered a marker of activation in sensorimotor and visual–spatial associative areas. In fMRI, Saleh et al. [31] studied the brain reorganization patterns of patients after VR combined with robot-assisted training and found that the hemispheric lateralization index values showed a downward trend, reflecting the reduction in initial contralateral hemispheric dominance after stroke, consistent with the promotion of functional recovery in the damaged hemisphere of stroke patients under the combined intervention of VR and rehabilitation robots [66].

#### 4.2.2. Adaptive Feedback and Reinforcement Learning

Reinforcement learning is a reward-driven learning paradigm and one of the most commonly used learning mechanisms in VR rehabilitation training. For precision rehabilitation, the goal of intensive learning is to obtain personalized treatment strategies that maximize rewards [67]. Reinforcement learning calibrates and customizes the task difficulty via game scoring, level unlocking, real-time positive feedback (e.g., the “excellent completion” prompt) and other reward mechanisms. Combined with the immersive environment created by VR [68], it establishes a closed loop of “training behavior–feedback reward-behavior optimization”. From the neurobiological model, this VR reward structure can be associated with the plasticity mechanism: a landing reward activates the related striatum, which promotes motor learning skills by regulating the release of dopamine [69]. Compared with error-based learning logic, VR stroke rehabilitation research tends to adopt a reinforcement learning mechanism, strengthen correct rehabilitation behavior through positive rewards, reduce patients’ resistance to training, and optimize the rehabilitation effect (also, there is no good punishment at present).

(1) Biofeedback

Research indicates that embedding biofeedback into virtual reality environments provides a feasible solution to address the challenges of biofeedback rehabilitation effects in terms of perception, autonomy, mastery, learnability, and motivation [41]. This mainly involves tactile feedback and visual feedback. In terms of tactile feedback, Bae et al. [27] developed a VR-based hand rehabilitation system with vibratory tactile feedback. fNIRS detection results showed that multisensory feedback stimulation activates the somatosensory association cortex, which is manifested as an increased hemodynamic response of oxygenated hemoglobin in the somatosensory association cortex. In terms of visual feedback, the representative approach is VR-based mirror therapy. Tunik et al. [32] found through fMRI analysis that the detection data from five stroke patients all showed that VR-based mirror visual feedback significantly activated the motor cortex of the damaged hemisphere during unilateral hand movement of the non-paralyzed hand, verifying that the neural mechanism behind mirror feedback promotes the development of specific brain network activity, thereby enhancing brain reorganization. To further explore the efficacy of bimanual movement patterns, Patricia et al. [33] compared the potential of mirror therapy involving both unilateral and bimanual movement patterns in a virtual environment. Electromyography results indicated that combining proprioceptive feedback from functional limbs may increase patient motivation and enhance the perception of greater usability of the damaged limb.

(2) Computer-Assisted Cognitive Rehabilitation

In advanced VR systems, multiple studies have emphasized their excellent effectiveness in rehabilitation within computer-assisted rehabilitation environments [54]. For instance, training prospective memory tasks such as preparing coffee in a virtual kitchen or shopping in a virtual convenience store significantly improves cognitive functions dependent on the frontal lobe and enhances the neuroplasticity of working memory [70]. Furthermore, diversified VR programs combined with cognitive therapy can reactivate the brain’s neurotransmitter capabilities (such as the cholinergic and dopaminergic systems) [55], thereby improving attention and memory in stroke patients to some extent. For example, Feitosa et al. [34] analyzed the effect of computer-aided cognitive function improvement in patients with chronic stroke based on fMRI and clinical evaluation. They found that the experimental group mainly involved increased connectivity in the fronto-parietal network, basal ganglia, and cerebellum, which is believed to be related to the improvement of patients’ executive function and attention control.

#### 4.2.3. Neural Regulation and Excitability Modulation

(1) Transcranial Electrical/Magnetic Stimulation

Noninvasive brain stimulation, such as transcranial direct current stimulation (tDCS) and transcranial magnetic stimulation (TMS), has been shown to improve motor and cognitive function in stroke patients by stimulating neuroplasticity and motor learning effects when combined with VR [37,38]. For the combined application of VR and tDCS, Lee et al. [35] first elucidated the regulatory functions of anodic tDCS and cathodic tDCS on the excitability of the motor cortex. Through randomized controlled experiments, they preliminarily concluded that the cumulative effect of cathodic tDCS and VR can promote upper-limb function in stroke patients by regulating different pathways, such as sodium and calcium voltage-dependent channels and cortical reorganization. To further validate Lee et al.’s conclusion, Yao et al. [36] compared the combined effect of cathodic tDCS and VR with VR alone, and, based on the scale evaluation results, concluded that the complementary mechanism of tDCS and VR induces changes in cortical excitability by regulating the conductivity of sodium and calcium channels and the activity of N-methyl-D-aspartate (NMDA) receptors, further regulating cortical neuroplasticity. Regarding the combined efficacy of anodic tDCS and cathodic tDCS, Llorens et al. [37] applied VR-based combination therapy to post-stroke hemiplegic patients. The results showed that compared with conventional physical therapy, the combined intervention increased the excitability of the motor cortex during the learning process by inhibiting the excitability of cortical circuits, providing clinically significant improvements in both motor and sensory functions of patients. For the combined application of VR and TMS, Chauhan et al. [38] found that repeated TMS and VR combined interventions lead to increased levels of neurotransmitters such as dopamine and acetylcholine and enhanced activity of NMDA receptors through direct stimulation of target areas in the patient’s brain, promoting cortical excitability and neural remodeling. This has a similar principle to the combined rehabilitation effect of tDCS and VR.

## 5. Discussion

This article systematically reviews the application of virtual reality rehabilitation training systems in the upper-limb, lower-limb, and cognitive rehabilitation of stroke patients. Combining a number of neuroimaging techniques to analyze the correlation between the rehabilitation effect of VR combined with other technologies and neuroplasticity, the review discusses the internal neural mechanism of VR rehabilitation intervention while paying attention to the clinical outcome of rehabilitation.

(1) Limitations of VR rehabilitation

Existing studies have shown that VR has become a potential intervention in rehabilitation medicine. However, it should be clear that VR rehabilitation does not apply to all stroke patients, and its effect is scene-dependent. In some cases, poor rehabilitation outcomes may occur [71]. The inferior VR rehabilitation effect mainly happens in the following three types of clinical situations: First, patients with severe cognitive impairment or dementia. Such patients are often accompanied by executive dysfunction and cannot effectively understand the VR training logic and operation requirements. Individual adjustments of the VR training parameters and explanation of the interface content are needed before intervention. Otherwise, the rehabilitation effect will be seriously affected [72]. Second, patients with severe aphasia. At present, the application of VR in language rehabilitation mainly focuses on the correction and improvement of mild and moderate language disorders. For patients with severe aphasia, the inability to accurately understand instructions during VR training disrupts the process and thus significantly reduces the effectiveness of rehabilitation interventions [73]. Third, patients with neuropsychological or physical defects are unable to maintain treatment stability. For example, the flash and dynamic visual stimulation contained in the VR rehabilitation environment may pose a life threat to patients with photosensitive epilepsy, and such patients need to be strictly contraindicated from VR rehabilitation intervention [74].

(2) Analysis of the differences in treatment schemes

At present, according to the needs of clinical rehabilitation, the VR rehabilitation training system has not yet formed a set of complete and unified rehabilitation evaluation standards. Its optimal parameter setting and training scheme still need to be determined through systematic comparison and critical analysis of various influencing factors.

Among them, the sense of immersion is one of the core factors affecting the effect of VR rehabilitation. The existing VR rehabilitation training system is mainly divided into two types: immersive and non-immersive, which have their own advantages and disadvantages in the application. The immersive VR system builds a closed and complete virtual environment for patients through helmets and other devices, which significantly improves the patient’s sense of scene presence and substitution, and better realizes human–computer interaction [75], but it is expensive and may cause visual discomfort. The non-immersive VR system has the advantages of simple equipment, low cost, and convenient operation, but its sense of immersion is weak, and the attraction to patients is limited, which may affect the training compliance and rehabilitation effect. It is vital to point out that there is no clinical trial evidence to directly compare the rehabilitation effect of immersive and non-immersive VR paradigms.

In addition to the types of VR systems, treatment schemes (such as training intensity, duration, frequency, and training mode) are also the key variables affecting the outcome of VR rehabilitation. The subgroup analysis results of Fang et al. [76] showed that more than 18 treatments can bring greater benefits to upper-limb rehabilitation; the meta-analysis of Gao et al. [77] verified that higher doses of VR rehabilitation intervention (more than 20 h of intervention) and higher frequencies (more than four times a week) elevated the recovery of cognitive and motor functions. However, although the rehabilitation results of stroke have revealed a positive correlation between treatment time and treatment results, the optimal daily intensity and weekly frequency of VR intervention are not clear. In order to promote the clinical standardization of VR rehabilitation technology, further research should be carried out in combination with the individual clinical conditions of patients.

In addition, the impact of patients’ clinical stage on VR rehabilitation effect should also be fully valued and analyzed. VR interventions are employed at the late stage of stroke recovery (more than six months) in many studies. The reason is not clear. The difference in the design level of this study may interfere with the evaluation of the rehabilitation effect, which needs to be analyzed in the follow-up study.

(3) Interpretation and limitation analysis of the neuroplasticity mechanism

As previously described, VR rehabilitation can promote the neuroplasticity of stroke patients through task-oriented training, adaptive feedback, neural regulation, and other methods, while the activation and functional reorganization of the cerebral cortex can be observed through neuroimaging technology. These changes are related to the effect of VR rehabilitation, but it should be clear that this correlation is not equivalent to causality.

Most of the existing studies have only observed the coexistence of “enhanced activation of a brain region after VR training” and “improved rehabilitation effect” through neuroimaging technology but have not yet proved the causal relationship between the two through strict intervention experiments, which is also one of the core limitations of the current neuroplasticity association research. Further analysis has found that the existing research on the correlation mechanism between VR and neuroplasticity in stroke rehabilitation is mostly based on small sample sizes and exploratory pilot studies and lacks large-sample and long-term follow-up confirmatory studies, leading to the lack of reliability and universality of the research conclusions. For example, some studies have used fMRI to observe the enhanced activation of the motor cortex in stroke patients after VR training. However, due to the small sample size (mostly 10–20 patients) and the influence of individual differences (such as age, underlying disease, injury location, and injury degree) not being excluded, it is difficult to determine whether this enhanced activation is the result of neuroplasticity changes directly caused by VR training or the combined effect of other factors (such as natural recovery, conventional rehabilitation training, and individual compensation mechanisms).

In addition, neuroimaging research also faces the following problems: First, large individual differences. Different stroke patients have different injury sites, injury degrees, and rehabilitation bases, which lead to significant differences in the changes in neuroimaging indicators after VR training. It is also difficult to form a unified judgment standard and evaluation system. Second, the problem of motion sensitivity. The head and limb movements of patients during VR training may lead to the distortion of neuroimaging detection signals, affecting the accuracy and reliability of the detection results. Third, the difficulty of clinical promotion. The existing neuroimaging technology is mostly suitable for scientific research scenarios. The equipment cost is high, and the operation is complex, which makes it difficult to popularize in primary medical institutions.

(4) Prospects for future research

On the whole, VR, through its three core characteristics of immersion, interactivity, and imagination, can promote the function of stroke patients in many aspects, induce the reorganization of brain structure and function, and promote neuroplasticity. It shows that VR is not only a rehabilitation tool but also an effective rehabilitation strategy that can actively regulate the neural circuit and promote the reconstruction of brain function.

In view of the above shortcomings, future research directions should focus on the following four aspects: First, carrying out large-sample, long-term follow-up randomized controlled trials, including positive and negative results, to identify the influencing factors of poor VR rehabilitation effect and establishing a complete evaluation system of VR rehabilitation effect. Second, strengthening critical and comparative research and optimizing the treatment scheme of VR rehabilitation (training intensity, duration, frequency, and training mode). Focus should be placed on formulating personalized rehabilitation plans according to the clinical stages and individual needs of patients so as to improve the pertinence and effectiveness of VR rehabilitation. Third, multimodal neuroimaging technology and strict intervention experiments should be used to clarify the causal relationship between VR training and neuroplasticity changes to improve the theoretical system of the correlation mechanism of VR rehabilitation, thus promoting neuroplasticity. Fourth, more focus should be put on promoting the clinical transformation and popularization of VR rehabilitation technology. We should develop low-cost, portable, and convenient VR rehabilitation equipment to improve the application feasibility for primary medical institutions and to let VR technology better serve the rehabilitation treatment of stroke patients and help the development of stroke rehabilitation.

## Figures and Tables

**Table 1:** Neuroimaging analysis of virtual reality rehabilitation training in stroke patients.

Plasticity	VR Modality	Rehabilitation Program	Neuroimaging Techniques	Baseline (HS: Healthy Subjects; SS: Stroke Survivor)			Targeted Functional Domain	Main Results		Conclusion	Study (First Author, Year)
				HS/SS	Sex F/M	Average		Clinical Results	Neuroimaging Results		
Task-oriented Training and Functional Reorganization	Interactive Game	Gesture-controlled rhythm game with vibrotactile feedback: 20 s, 10 times	fNIRS, 10 times	11/1	-	25.4 ± 4.4, 22	PFC, PMC&SMA, SM1, SAC ^1^	The success rate of gesture matching: HS (90% ± 10.7%); SS (79.6%)	Significant activation in SM1, PMC&SMA, PFC, and SAC; 19/51 channel HbO concentration increase.	VR rhythm games can enhance the activation of motor-related brain regions and promote neuroplasticity.	Bae et al. (2023) [27]
		The Kinect-based virtual reality system	fMRI, 3 times	18/5	-	-	SM1, SMA, Cerebellum	FMA (54.20 ± 4.43); WMFT (3.80 ± 0.35) ^2^	Primary sensorimotor cortex activation areas increased for patients 1–4, while for patient 5, it gradually decreased over all three time points.	Kinect-VR can enhance the activation of contralateral S1/M1.	Bao et al. (2013) [28]
		VR-based bilateral upper-extremity training: 30 min/day, 3 times/week, for 6 consecutive weeks	EEG, 1 time	0/18	8/10	-	Frontopolar1, 2, 3, 4	-	The concentration of EEG activity in frontopolar 2 and frontal 4 increased significantly in the VR group (*p* < 0.01).	VR bilateral upper-limb training can enhance the brain activity of the frontal central region.	Lee et al. (2015) [29]
	Rehabilitation Robot	RAGT ^3^ + VR: 45 min/time, 5 times/week, for 8 consecutive weeks	EEG, 2 times	0/24	14/10	60 ± 4; 63 ± 6	Premotor, precuneus, and associative visual areas	RMI 14 ± 1; POMA 23 ± 3; VAS 8 ± 1 ^4^	Hγ-ERD (t = 4, *p* < 0.001); β-ERD (t = −26, *p* < 0.001); μ/β-ERSP (r = 0.895/0.570, *p* < 0.05). ^5^	Using VR in RAGT can enhance the activation of motor-related brain regions and the modulation of EEG oscillations.	Calabrò et al. (2017) [30]
		Robot-assisted arm and hand training in VR: 3 h/day, for 8 consecutive days	fMRI, 3 times	0/4	2/2	61.5 ± 7	The ipsilesional motor cortex (precentral gyrus)	The average improvement of WMFT is about 20%, and the average improvement of JHFT ^6^ is about 25%	All 4 patients’ functional connection between the affected motor cortex and the bilateral sensorimotor cortex was significantly enhanced. The activation of the sensorimotor cortex increased in 2 and decreased in 2.	Robot-assisted VR training can enhance the recruitment of the sensory motor cortex on the affected side and change the balance between hemispheres.	Saleh et al. (2011) [31]
Adaptive Feedback and Reinforcement Learning	Biofeedback	Gesture-controlled rhythm game with vibrotactile feedback; 20 s; 10 times	fNIRS, 10 times	11/1	-	25.4 ± 4.4, 22	PFC, PMC&SMA, SM1, SAC ^1^	The success rate of gesture matching: HS (90% ± 10.7%); SS (79.6%)	Significant activation in SM1, PMC&SMA, PFC, and SAC; 19/51 channel HBO concentration increase.	VR rhythm games can enhance the activation of motor-related brain regions and promote neuroplasticity.	Bae et al. (2023) [27]
		Mirror-visual feedback presented in VR: 2 experiments, 8 trials, 4 runs/subject	fMRI, each inter-trial rest period	0/5	3/2	56.6 ± 12	Ipsilesional sensorimotor cortex	-	Significant activation was noted in the sensorimotor system of the ipsilesional hemisphere (ipsilateral to the moving hand) (*p* < 0.01).	Mirror visual feedback in VR games during unimanual motion of the unaffected hand can significantly activate the motor cortex of the lesioned hemisphere.	Tunik et al. (2011) [32]
		VR-based Mirror Visual Feedback: 2 times/day	sEMG, 8 sEMG sensors	18/0	14/4	-	The anterior (flexor) and posterior (extensor) compartments of the forearm	Visual feedback type: BBT (*p* = 0.16); TPD (*p* = 0.076); APD (*p* = 0.80414) ^7^	-	VR-based physical rehabilitation can augment multisensory real-time feedback to promote neuroplasticity.	Patricia et al. (2024) * [33]
	Computer-assisted Cognitive Rehabilitation	The GestureCollection VR-based rehabilitation: 2 times/week for 8 consecutive weeks	fMRI, 2 times	0/10	-	-	The frontoparietal and the somatomotor networks, cerebellum, and basal ganglia	RMV (%): FMA (12.7, 8.5, 7); BBS (12); TUG (−16); MoCA (11) ^8^	Increases in the range (50, 75%): RFPtc, in CC; and LFPtc, LNAC, and RNAC, in S. ^9^	VR performed with GestureCollection can enhance the functional connection of reward-related motor learning networks (frontal-parietal somatic movement, cerebellum-basal ganglia).	Feitosa et al. (2023) [34]
Neural Regulation and Excitability Modulation	Transcranial Electrical/Magnetic Stimulation	c-tDCS combined with VR: 15 times in 3 weeks	-	0/59	-	-	-	Group C (received combination therapy): ∆MAS (0.1 ± 0.5); ∆MFT (4.7 ± 3.3) ^10^	-	A combination of tDCS and VR therapy leads to greater recovery of upper-limb impairment than each intervention alone in patients with subacute stroke.	Lee et al. (2014) * [35]
		c-tDCS combined with VR: 20 min/day, 10 times in total	-	0/40	31/9	-	-	FMA (34.4 ± 17.8); ARAT (24.8 ± 19.9); BI (72.0 ± 17.1) ^11^	-	c-tDCS positioned over M1 of the unaffected hemisphere, combined with VR therapy, can reduce ipsilateral upper-limb motor impairment, improve motor function, and enhance ADL of the patient.	Yao et al. (2020) * [36]
		A combined tDCS and VR-based intervention: 1 h/time, 25 times in total	-	0/29	22/7	54.9 ± 9.4	-	FMA (*p* < 0.001, η^2^*_p_* = 0.44); WMFT (*p* = 0.036, η^2^*_p_* = 0.15), the ability subscales (*p* = 0.043, η^2^*_p_* = 0.14)	-	VR combined with tDCS can reduce cortical inhibitory circuit excitability during practice, thereby enhancing motor cortex excitability in motor learning.	Llorens et al. (2021) * [37]
		Integrating VR and TMS: 12 lessons in 3 weeks	-	0/69	-	-	-	*p*-Value: memory 0.009; language 0.01; ACE III ^12^ 0.004	-	After rTMS and VR treatment, γ—aminobutyric acid-mediated inhibition and N-methyl-D-aspartate receptor activity increase, cortical excitability is significantly changed, and the response ability of cortical neurons to stimulation is improved.	Chauhan et al. (2024) * [38]

Note: * indicates that the experiment does not involve three neuroimaging techniques. 1. The prefrontal cortex (PFC), the premotor cortex and the supplementary motor area (PMC&SMA), the primary sensorimotor cortex (SM1), and the somatosensory association cortex (SAC). 2. Fugl-Meyer Assessment (FMA) and Wolf Motor Function Test (WMFT) are used to evaluate the recovery of motor function in stroke patients. 3. RAGT—mechanical and electrical equipment that can provide external support to the patient’s limbs and make the affected limb produce normal movement during walking. 4. Rivermead mobility index (RMI)—a standardized assessment tool widely used in the field of rehabilitation medicine, which is mainly used to assess the recovery of patients’ motor function. The Tinetti Performance Oriented Mobility Assessment (POMA)—used to evaluate individual’s activity and balance ability. 5. Event-related desynchronization (ERD)—used to describe the phenomenon that the oscillation power of neurons in the cerebral cortex decreases in a specific task. Event-related spectral perturbation (ERSP)—analyzes the changes in neural oscillations in the brain under specific tasks or stimuli. 6. Jebsen hand function test (JHFT)—mainly used to assess the ability of daily living of the hand. 7. Practice effects on Box and Blocks Test (BBT); Time per Disk (TpD); and Accuracy per Disk (ApD). 8. Relative mean variation (RMV); the Berg Balance Scale (BBS)—used to assess balance; the Timed Up and Go (TUG)—a test of mobility, gait, and balance; and the Montreal Cognitive Assessment (MoCA)—an effective method for cognitive assessment. 9. The right frontoparietal task control region (RFPtc), the left frontoparietal task control (LFPtc), the left nucleus accumbens (LNAC), the right nucleus accumbens (RNAC), the strength (S), and the clustering coefficient (CC). 10. The Modified Ashworth Scale (MAS)—standardized tools for assessing muscle spasm; Manual Function Test (MFT)—evaluates unilateral manual performance in hemiparetic patients after stroke. 11. The Action Research Arm Test (ARAT) and the Barthel Index (BI). 12. Addenbrooke’s Cognitive Examination III (ACE III)—a comprehensive screening examination that assesses an individual’s cognitive abilities in multiple domains.

## Data Availability

No new data were created or analyzed in this study.
